# Improvements of Electrical Characteristics in Poly-Si Nanowires Thin-Film Transistors with External Connection of a BiFeO_3_ Capacitor

**DOI:** 10.3390/membranes11100758

**Published:** 2021-09-30

**Authors:** Tsung-Kuei Kang, Yu-Yu Lin, Han-Wen Liu, Che-Li Lin, Po-Jui Chang, Ming-Cheng Kao, Hone-Zern Chen

**Affiliations:** 1 Department of Electronic Engineering, Feng Chia University, Taichung 407, Taiwan; zjwy0507@gmail.com (Y.-Y.L.); jackyjoy551709@gmail.com (C.-L.L.); 2 Department of Electrical Engineering, National Chung Hsing University, Taichung 407, Taiwan; hwliu@dragon.nchu.edu.tw (H.-W.L.); z387253@gmail.com (P.-J.C.); 3 Department of Electrical Engineering, Hsiuping University of Science and Technology, Taichung 407, Taiwan; kmc@hust.edu.tw (M.-C.K.); hzc@hust.edu.tw (H.-Z.C.)

**Keywords:** BiFeO_3_ capacitor, sol–gel method, drive current, hysteresis, subthreshold swing, incomplete dipole flipping

## Abstract

By a sol–gel method, a BiFeO_3_ (BFO) capacitor is fabricated and connected with the control thin film transistor (TFT). Compared with a control thin-film transistor, the proposed BFO TFT achieves 56% drive current enhancement and 7–28% subthreshold swing (SS) reduction. Moreover, the effect of the proposed BiFeO_3_ capacitor on I_DS_-V_GS_ hysteresis in the BFO TFT is 0.1–0.2 V. Because dV_int_/dV_GS_ > 1 is obtained at a wide range of V_GS_, it reveals that the incomplete dipole flipping is a major mechanism to obtain improved SS and a small hysteresis effect in the BFO TFT. Experimental results indicate that sol-gel BFO TFT is a potential candidate for digital application.

## 1. Introduction

Recently, negative capacitance (NC) transistors have been widely studied, because they are considered to be one of the most promising candidates for low power applications. The NC transistor is first proposed by Salahuddin and Datta. In NC transistors, an embedded ferroelectric film in the gate stack plays a role of voltage amplification [1]. Many studies have been demonstrated transistor integrated with various ferroelectrics, such as HfZrOx [2,3,4,5,6,7], PbZrTiO_3_ [8,9], PVDF [10,11], and BiFeO_3_ (BFO) [12]. NC transistors are expected to be applied for logic switching, which requires hysteresis-free characteristics. Generally, the phenomena of clockwise hysteresis show their NC effect. Some studies reveal that a complete dipole flipping in ferroelectric can obtain a large hysteresis, but an incomplete dipole flipping in ferroelectric can obtain a small hysteresis [13,14,15]. BFO is a single phase multiferroic material with a rhombohedrally distorted perovskite with polar space group R3c at room temperature [16]. Although BFO is a very good ferroelectric material, it is still not widely used in negative capacitance transistors. In reference [12], although BFO negative capacitance transistors can achieve a very low subthreshold swing (SS) from 8.5 to 50 mV/decade, the effect of BFO capacitor on the hysteresis is about 4–5 V. Therefore, as far as digital applications are concerned, more studies are needed for BFO negative capacitance transistors. In this paper, BFO thin film on Pt will be fabricated by a low-cost sol–gel method [17] and the Poly-Si nanowire junctionless thin film transistor (TFT) externally connected to the BFO capacitor will be investigated. Interestingly, our results reveal that the incomplete dipole flipping in the BFO capacitor plays a major role to obtain a small hysteresis effect in the proposed BFO TFT device.

## 2. Devices Fabrication

**Figure 1:**
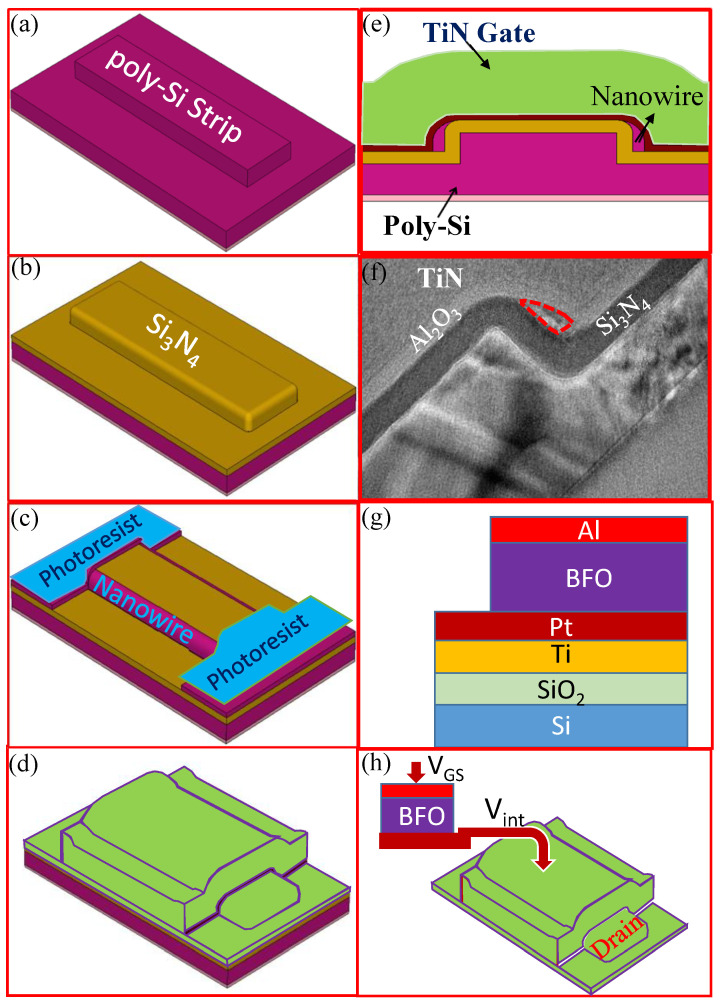
Key fabrication steps for the control TFT and BFO capacitor. (**a**) N^+^ Poly-Si layer deposition and strip patterning, (**b**) Si_3_N_4_ deposition, (**c**) N^+^ Poly-Si layer deposition, N^+^ Poly-Si NW channels and source/drain were Figure 2. (**d**) O_3_ gate oxide and TiN deposition, then gate patterning, (**e**) the cross-sectional transistor with TiN gate, (**f**) the cross-sectional TEM image, where the red line indicates the Si channel. Its peripheries are 9.4 nm, 24 nm, and 35 nm, respectively. Due to 5-nm-thick Al_2_O_3_, the effective width (Weff)/channel of TiN gate is about 30nm in control TFT. (**g**) Schematic diagram of BFO capacitor. (**h**) A wire is connected to the BFO capacitor and a control transistor where it can be measured.

A 100-nm-thick in-situ phosphorus-doped (N^+^) Poly-Si was deposited on oxidized silicon wafer by low-pressure chemical vapor deposition (LPCVD). The 100-nm-thick Poly-Si was then patterned, using partial etching to form one 50-nm-thick Poly-Si strip, as shown in Figure 1a. Then, a 20-nm-thick Si_3_N_4_ film was deposited by LPCVD, as shown in Figure 1b. Next, a 15-nm-thick N+ Poly-Si was deposited by LPCVD at 560 °C and then annealed at 600 °C for 10 hours, which recrystallized it into Poly-Si through solid-phase crystallization. Then, using standard I-line lithography, the photoresists on the source/drain (S/D) pads were patterned to overlap on the two edges of the raised strip. After that, a plasma etcher was used to remove the N^+^ Poly-Si, while the spacer Si channels were formed on the sidewall of each L-type Si_3_N_4_ film, in situ, and naturally connected to the S/D pads, as shown in Figure 1c. Next, S/D-pad photoresists were removed and 5-nm-thick atomic layer deposition (ALD). Al_2_O_3_ and 100-nm-thick TiN were deposited by sputtering system as the gate oxide and the gate electrode, as shown in Figure 1d. The schematic cross-sectional channel and its TEM image were shown in Figure 1e,f. In this paper, the main purpose is to study the influence of BiFeO_3_ (BFO) capacitor on the characteristics of transistors. Therefore, the process of control transistors used in the paper is not optimized.

For BiFeO_3_ capacitors, BFO thin film was fabricated on Pt/Ti/SiO_2_/Si by a sol–gel method. Bismuth (III) acetate [Bi(OOCCH_3_)_3_] and Iron(III) 2,4-pentanedionate [C_15_H_21_FeO_6_] were used as raw materials. These starting materials were first dissolved in propionic acid and 2-methoxyethanol with Bi excess 5%. The solution was then stirred at 80 °C for 4h to obtain a uniform sol solution, and was subsequently coated in Pt/Ti/SiO_2_/Si substrate with 2500 rpm for 30 s and dried at 300 °C for 2 min. This step was repeated 10 times to obtain the final BFO films (~300 nm). The BFO film was annealed at 550 °C for 30s and called 550-BFO. Finally, Al were deposited by sputtering as with the top gate, as shown in Figure 1g. A wire was connected to the 550-BFO capacitor and control TFT, as shown in Figure 1h. All upper electrodes were circular, and a BFO capacitor with a radius of 170 μm was used in the study of the NC effect in the resistive–capacitive (RC) circuit, and the BFO capacitor with a radius of 28um was connected in series with the control transistor. The size of the control TFT is Weff = 30 nm × 2/L = 5 μm in the proposed 550-BFO TFT.

## 3. Results and Discussion

**Figure 2:**
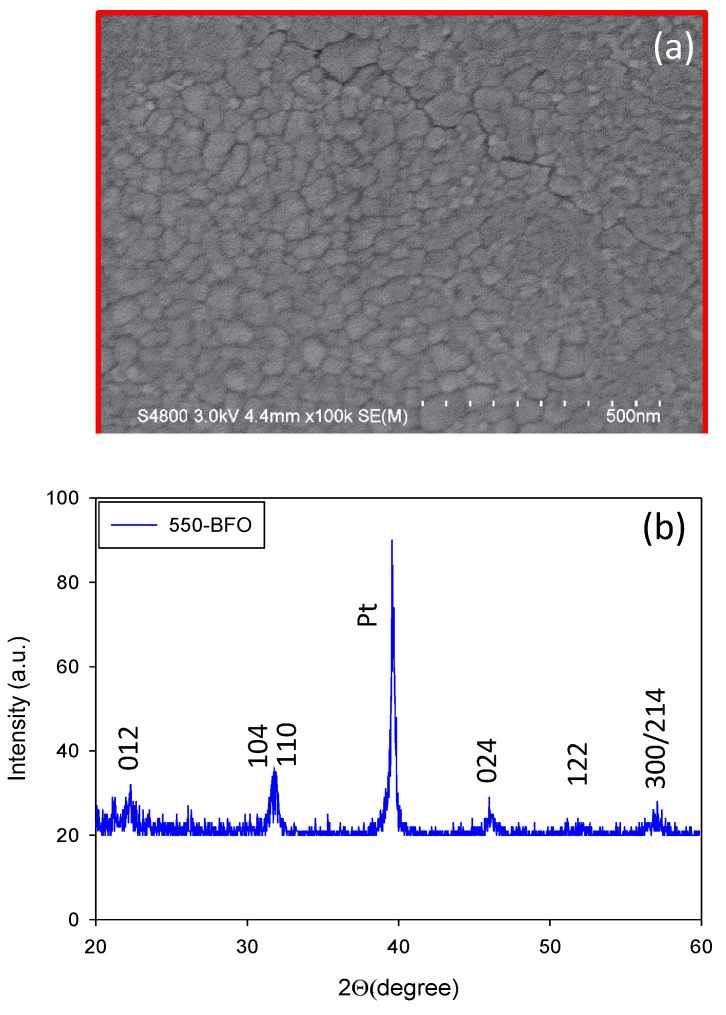
(**a**) SEM image of BFO film annealed at 550 °C. (**b**) XRD pattern of BFO film annealed at 550 °C.

For BFO film, the grain structure of the film was detected using a scanning electron microscope (SEM), as shown in Figure 2a. The 550-BFO film shows relatively obvious grains. It reveals that the BFO film annealed at 550 °C is well crystallized. XRD patterns of the BFO film annealed at 550 °C is shown in Figure 2b and the (012), (110), (104), (024), (122), (300), and (214) diffraction peaks can be observed. The XRD peaks are quite similar to those of standard diffraction patterns of pure BFO on the joint committee on powder diffraction standards (JCPDS #71-2494) card. This indicates the formation of pure BFO phase, good crystallization, and rhombohedrally-distorted perovskite crystal structure with space group R3c [18]. According to the previous study, it reveals that the proposed 550-BFO film has good ferroelectric properties [18].

**Figure 3:**
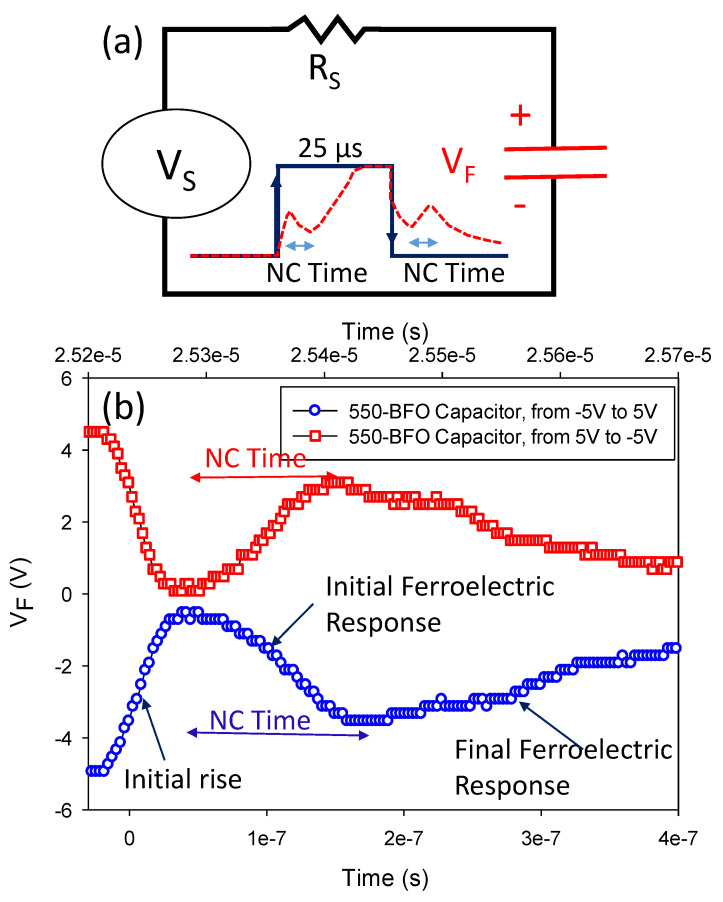
(**a**) Schematic diagram of experimental setup where the BFO capacitor is connected in series with a resistor (RC circuit). The pulse time is 25 μs. In the zoom, the solid line represents the input Vs signal and the dotted line represents the output voltage of the BFO capacitor. (**b**) Transient response of V_F_ for BFO capacitor. The NC time is 0.160 μs from −5 V to 5 V and 0.136 μs from 5 V to −5 V.

To study the NC effect, we created an RC circuit diagram of the experimental setup where the 550-BFO capacitor is connected in series with an external resistor Rs = 500 Ω, as shown in Figure 3a. An AC voltage pulse sequence of Vs: −5V → +5V → −5V was applied as input and the voltage (V_F_) across the BFO capacitor was recorded by an oscilloscope. Figure 3b shows the transient response of V_F_ from −5V to +5V and from +5V to −5V for the 550-BFO capacitor. In Figure 3b, the spike behavior can be understood in the following sequence: initial rise, initial ferroelectric response, and final ferroelectric response. According to reference [12], the NC time is defined the elapsed time from the highest value (or lowest value) to the lowest value (or highest value) of V_F_ in “initial ferroelectric response” and a longer NC time shows a longer charge compensation time after domain switching. Obviously, for the positive domain switching, the NC time is 0.160 μs and 0.136 μs for the negative domain switching. Therefore, whether with forward- or reverse sweeping, it is expected that the characteristics of 550-BFO TFT will be improved over the control TFT. 

**Figure 4:**
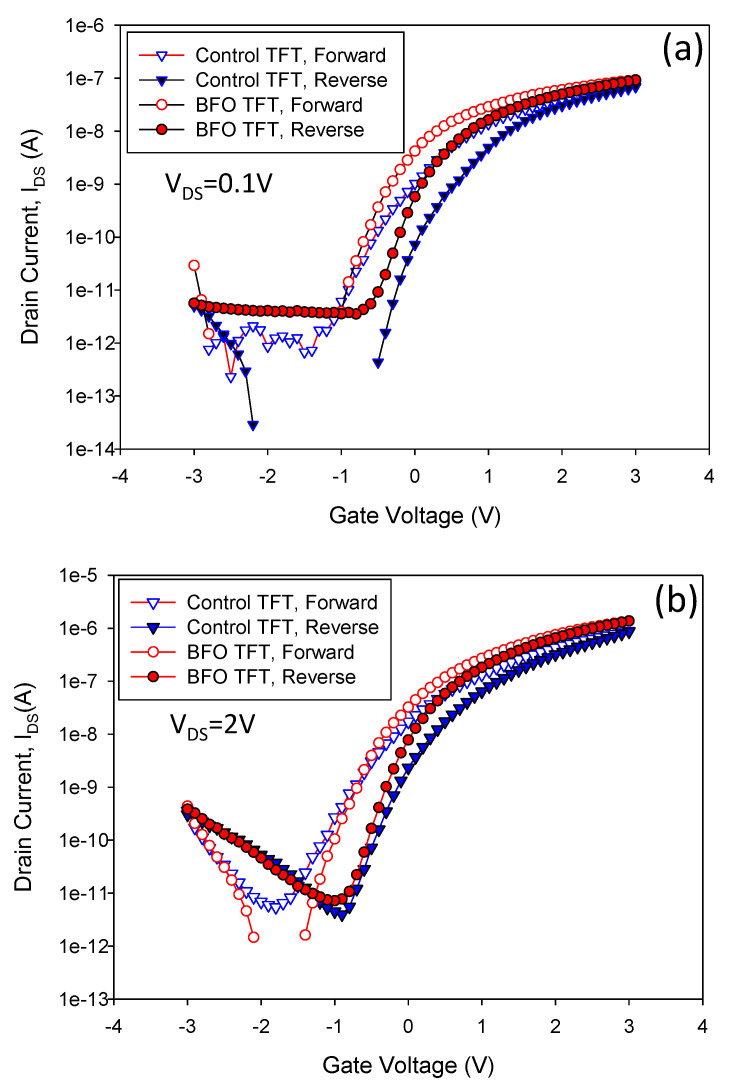
(**a**) I_DS_-V_GS_ transfer characteristics at V_DS_ of 0.1 V for control TFT (L = 5 μm, Weff = 60 nm) and 550-BFO TFT. (**b**) I_DS_-V_GS_ transfer characteristics at V_DS_ of 2 V for control TFT and 550-BFO TFT.

The I_DS_-V_GS_ transfer characteristics at V_DS_ of 0.1 V and 2 V for control TFT and 550-BFO TFT are shown in Figure 4b, respectively. The V_TH_ is defined as the gate voltage that is required to obtain a normalized drain current of I_DS_ = (W_eff_/L) × 10^−8^ A. For 550-BFO TFT, the hysteresis from 0.59 V to 0.45 V at V_DS_ of 0.1 V is observed, as shown in Figure 4a. At V_DS_ of 2 V, the hysteresis from 0.6 V to 0.4 V is observed, as shown in Figure 4b. From the hysteresis curves, the effect of the 550-BFO capacitor on hysteresis is about 0.14 V and 0.2 V at 0.1 V and V_DS_ of 2 V, respectively. For 550-BFO TFT, no matter at V_DS_ of 0.1 V or 2 V, the ON current (I_ON_) at V_GS_ of 3 V shows 56% enhancement over control TFT. 

**Figure 5:**
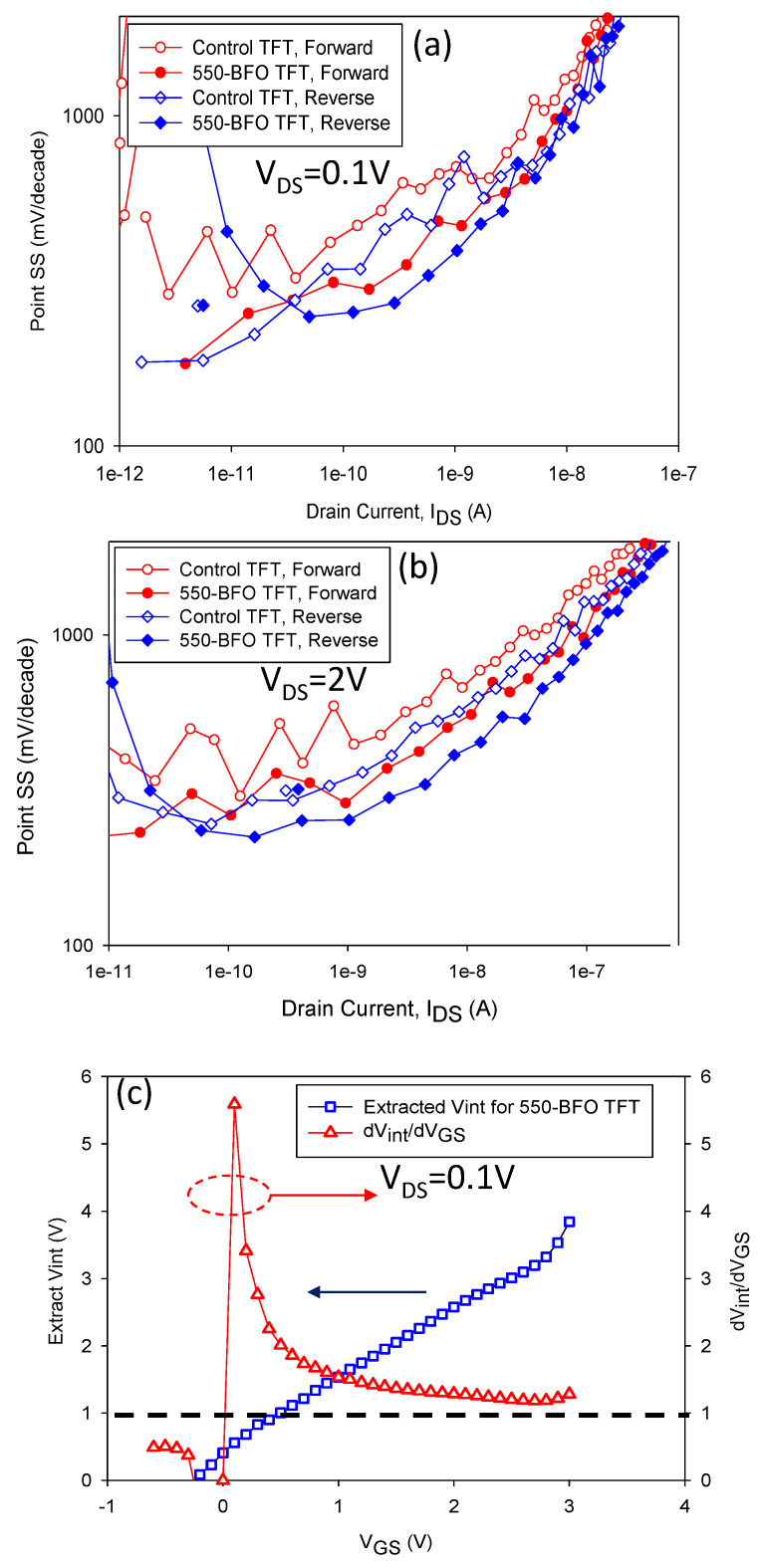
Point SS versus I_DS_ curves for (**a**) control TFT (L = 5 μm, Weff = 60 nm) and 550-BFO TFT at V_DS_ of 0.1 V and (**b**) at V_DS_ of 2 V. (**c**) Extracted Vint and dVint/dV_GS_ versus V_GS_ at V_DS_ of 0.1 V. The position of V_GS_ and V_int_ are marked in Figure 1h.

Figure 5a,b shows the point SS versus I_DS_ curves for control TFT and 550-BFO TFT. Compared with control TFT, no matter forward sweeping or reverse sweeping, 550-BFO TFT show improved SS characteristics at V_DS_ of 0.1 V or 2 V over control TFT. In the I_DS_ range of 1 × 10^−^^8^ to 1 × 10^−^^11^ A, the average SS value is reduced by 28% for forward sweeping and 7% for reverse sweeping. The SS data is consistent with the trend of NC time of positive and negative domain switching. Based on the assumption that the I_DS_ of 550-BFO TFT is the same as the control TFT, the extracted V_int_-V_GS_ curve at V_DS_ of 0.1V can be obtained [15]. dV_int_/dV_GS_ versus V_GS_ can be calculated, as shown in Figure 5c. It is found that dV_int_/dV_GS_ > 1 is obtained at a wide range of V_GS_, leading to the improved SS over control TFT in the whole measuring range of I_DS_ in 550-BFO TFT. The previous study reported that the mechanism underlying near I_DS_-V_GS_ hysteresis-free transistor is incomplete dipoles flipping, rather than complete dipoles switching in the transistor with a large I_DS_-V_GS_ hysteresis [15]. Therefore, the incomplete dipole flipping plays a major role to obtain improved SS and a small hysteresis effect in the proposed 550-BFO TFT. According to the data and discussion mentioned above, it reveals that sol-gel BFO TFT is a potential candidate for digital application.

## 4. Conclusions

The proposed BFO TFT shows improved characteristics of I_ON_ increased by 56% and SS reduced by 7–28%, because the sol gel BFO film show good crystallization, ferroelectric property, and a long enough NC time. Based on the extracted V_int_-V_GS_ curve, dV_int_/dV_GS_ > 1 is obtained at a wide range of V_GS._ Obviously, the incomplete dipole flipping plays a major role in the proposed BFO TFT. Therefore, the effect of the proposed BFO capacitor on I_DS_-V_GS_ hysteresis in the BFO TFT is small. It reveals that the proposed BFO TFT is a potential candidate for digital application.

## Data Availability

Not applicable.
